# The Cusp-Overlap Technique Versus the Standard Three-Cusp Technique for Self-Expanding Transcatheter Aortic Valves—A Single-Center Experience

**DOI:** 10.3390/jcm15197413

**Published:** 2026-09-24

**Authors:** Alexandru Ispas, Damien Brunet, Redouane Saady, Yacoub Habib, Catalin Pestrea, Laurent Drogoul

**Affiliations:** 1 Department of Cardiology, Monaco Cardiothoracic Center, 98000 Monaco-Ville, Monaco; 2 Department of Cardiology, Brașov Regional Emergency Hospital, 500024 Brașov, Romania; pestrea.catalin@gmail.com

**Keywords:** transcatheter aortic valve replacement, self-expanding valve, cusp-overlap technique, permanent pacemaker implantation

## Abstract

**Background:** Conduction disturbances and the subsequent need for permanent pacemaker implantation (PPMI) remain a major limitation of transcatheter aortic valve replacement (TAVR) with self-expanding transcatheter heart valves (SE-THVs). The cusp-overlap technique (COT) has been proposed to optimize implantation depth and reduce PPMI rates compared to the standard three-cusp technique (3CT). **Methods:** This retrospective, single-center study included 113 consecutive patients with symptomatic severe aortic stenosis who underwent TAVR with the Medtronic CoreValve Evolut™ SE-THV(Medtronic, Minneapolis, MN, USA) between September 2020 and March 2023. Patients were treated using either COT (*n* = 49) or 3CT (n = 62). Baseline characteristics, procedural outcomes, and 30-day clinical events were compared before and after one-to-one propensity score matching. The primary endpoint was the incidence of PPMI at hospital discharge; the secondary endpoint was a composite of death, stroke, life-threatening or disabling bleeding, major vascular complications, and moderate/severe paravalvular regurgitation at 30 days. A post hoc power calculation was performed given the low observed event rate. **Results:** Procedural success was achieved in 98.2% of patients. In the unmatched cohort, PPMI occurred in 6.1% (COT) vs. 9.7% (3CT) (*p* = 0.48). In the matched cohort (49 pairs), PPMI rates were 6.1% (COT) vs. 12.2% (3CT) (*p* = 0.48). With only 3 vs. 6 PPMI events in the matched cohort, the study had an estimated post hoc power of approximately 19% (two-sided α = 0.05) to detect the observed difference, so the negative primary result should be regarded as inconclusive rather than as evidence of equivalence between techniques. The secondary composite endpoint occurred less frequently in the COT group both before matching (14.3% vs. 32.3%; *p* = 0.049) and after matching (14.3% vs. 30.6%, *p* = 0.049). This composite difference was driven predominantly by the moderate/severe paravalvular regurgitation (PVL) component (12.2% vs. 24.2% unmatched; 12.2% vs. 22.4% matched) rather than by death, stroke, or major vascular complications. Evolut device generation differed markedly by arm (COT patients received later-generation devices far more often than 3CT; *p* < 0.001), and is presented in the Methods/Results Sections as a key temporal confounder. COT was associated with a lower incidence of moderate/severe paravalvular regurgitation, without prolonging procedural time. **Conclusions:** In this real-world single-center cohort, the cusp-overlap technique was not associated with a statistically significant reduction in PPMI after TAVR with the CoreValve Evolut™ SE-THV. COT was, however, associated with fewer paravalvular leaks and a lower rate of the secondary composite of adverse clinical events at 30 days.

## 1. Introduction

Aortic stenosis is considered the most common valvular heart disease requiring cardiovascular surgery or transcatheter intervention [1]. The introduction of transcatheter aortic valve replacement (TAVR) has dramatically improved the treatment of symptomatic severe aortic valve stenosis in high-risk patients. TAVR is increasingly being used as an alternative to surgical aortic valve replacement across all surgical risk categories [2,3,4,5,6,7].

Nevertheless, post-TAVR conductive disorders, more often associated with self-expanding bioprostheses and mainly linked to the depth of implantation and/or the deployment technique, remain a major pitfall of the procedure. These complications increase the risk of morbidity and mortality and extend the duration of hospitalization [8,9,10,11,12,13].

Various methods have been suggested for determination of the optimal projection during TAVR. Self-expanding THVs engage the aortic valve from the outer aortic curvature and are deployed from the NCC toward the LCC. The cusp-overlap technique (COT) facilitates visualization of the real implantation depth and precise valve positioning during self-expanding THV implantation. Optimal projection is generated by overlapping the RCC with the LCC. The NCC is displayed in the center of the fluoroscopic image, allowing the operators to deploy the device relative to the membranous septum and the conduction system below the atrioventricular node [13,14,15,16].

Consequently, we designed this study to compare the clinical outcomes and the 30-day PPMI rates between the COT and the standard three-cusp technique (3CT) in patients undergoing TAVR with the SE Medtronic Evolut™ THV.

## 2. Methods

This is a retrospective, single-center observational study, conducted between September 2020 and March 2023 at the Cardio-Thoracic Center of Monaco. All patients with symptomatic severe aortic stenosis were analyzed by a multidisciplinary cardiac team, and the treatment modality was determined based on the most recent European guideline recommendations [1]. We identified 113 consecutive patients undergoing the TAVR procedure with the Evolut™ self-expanding valve.

Patient Exclusion Criteria: Out of 151 patients implanted with the SE-THV, 15 patients had a previously implanted pacemaker, 20 patients underwent a valve-in-valve procedure, and 3 patients had a non-femoral approach.

### 2.1. TAVR Planning and Procedure

Pre-TAVR computed tomography (CT) imaging was performed following standardized recommendations [17]. Post-processing was accomplished using dedicated software (EndoSize™ V4, Therenva, Rennes, France). The prosthesis size was chosen based on the perimeter of the aortic annulus. The COT view was determined on CT by superimposing the left and right coronary sinuses to isolate the non-coronary sinus. All procedures were performed in a hybrid cardiac catheterization room under general anesthesia. The Evolut generation used (classic/R, PRO, or PRO+) for each case was extracted from the procedural database. Because COT adoption increased over the study period and coincided with the introduction of later device generations (χ^2^ = 28.3, *p* < 0.001 for the generation-by-technique association), technique and device generation are strongly correlated with calendar time.

### 2.2. Data Collection and Monitoring

Baseline patient characteristics were collected in a clinical database. Electrocardiography was performed before the procedure, within the first 24 h, and before discharge. All patients received continuous electrocardiographic monitoring by telemetry for at least 24 h. In the presence of predictors of high-grade atrioventricular block, monitoring was extended to at least 48 h according to current expert consensus [18]. Baseline conduction parameters—pre-existing right bundle branch block, first-degree atrioventricular block, and baseline QRS/PR duration—were not consistently recorded for all patients in this retrospective database and are therefore not reported as a complete comparison between groups. Paravalvular regurgitation was assessed by transthoracic echocardiography (TTE) prior to hospital discharge and again at the 30-day follow-up visit, graded according to VARC-3 criteria [19] by the treating cardiologist at each time point; images were not adjudicated by a blinded core laboratory, and inter-observer variability in PVL grading is discussed as a limitation.

### 2.3. Endpoints

The primary endpoint was the incidence of permanent pacemaker implantation (PPMI) at hospital discharge. All patients were also seen at the routine 30-day follow-up visit, at which any pacemaker implantation occurring after discharge was captured; no patient in either group required PPMI between discharge and the 30-day visit in this cohort. Pacing indications followed the 2021 ESC guidelines [20]: high-degree/complete AV block, new-onset persistent LBBB with PR/HV prolongation, or alternating bundle branch block. The secondary endpoint was the composite of all-cause mortality or stroke, life-threatening or disabling bleeding, major vascular complications, and moderate or severe paravalvular leak (VARC-3 criteria [19]) at 30-day follow-up. Each component of this composite for which individual counts were available is additionally reported separately (Supplementary Materials, Table S2), since combining hard clinical events with an echocardiographic/imaging parameter is unconventional and the composite result is driven predominantly by the PVL component.

### 2.4. Statistical Analysis

Baseline, procedural, and in-hospital outcome characteristics were compared for both techniques and after one-to-one propensity score matching. The propensity score was estimated by logistic regression, including age, sex, STS risk score, renal insufficiency, coronary artery disease, hypertension, aortic annulus diameter, and NYHA class as covariates. Patients were matched 1:1 using nearest-neighbor matching without replacement and with a caliper width of 0.2 of the standard deviation of the logit of the propensity score. Pre-matching standardized mean differences (SMDs) for these covariates, computed directly from the study database, are reported in Supplementary Materials, Table S1, and confirm the expected imbalance in renal insufficiency and coronary artery disease that motivated matching. Post-matching SMDs could not be recomputed for this revision because the identities of the 49 matched pairs were not retained in the files available to the authors; only the aggregate matched-cohort results were returned by the statistician. Post-matching covariate balance is, therefore, evidenced only by the non-significant *p*-values already shown in Table S3, which is stated explicitly as a limitation. Quantitative data are expressed as median and 25–75th percentiles; qualitative data are expressed by frequency and percentage. Comparisons were made with the Mann–Whitney or chi-square test. A *p*-value < 0.05 was considered statistically significant. Given the low number of primary endpoint events, a post hoc power calculation was performed for the primary comparison (matched cohort) using the arcsine (Cohen’s h) approximation for two independent proportions; a formal a priori sample size calculation was not performed, which is acknowledged as a limitation. A temporal/sequential analysis of PPMI and PVL rates across the study period is presented in Section 3 to explore potential confounding from the operator learning curve and evolving device generation. Baseline conduction status (RBBB or first-degree AV block) could not be entered into the propensity model as it was not consistently available across the cohort (see the Study Limitations Section).

## 3. Results

A total of 113 patients underwent a TAVR procedure using a self-expanding bioprosthesis. In total, 43% (n = 49) were implanted using the cusp-overlap technique. The mean age was 84 years; 70 patients (62.83%) were female; and the average STS risk score was 8% (6; 12). No patient was lost to follow-up.

### 3.1. Unmatched Cohort—Baseline Characteristics

Baseline patient characteristics were similar between the groups, with renal insufficiency being significantly lower in the COT group (*p* = 0.027) (Table 1).

### 3.2. Unmatched Cohort—Procedural Data

Procedural success was achieved in 111 patients (98.23%). The right femoral artery was the preferred vascular access point in 92 patients (81.41%). Pre-dilatation was more often performed in the three-cusp group. Procedure time was shorter in the COT arm (Table 2). Device generation differed markedly by arm (Table 2): the 3CT group received predominantly earlier-generation devices (Evolut R: 32.3%, PRO: 50.0%, and PRO+: 17.7%) while the COT group received predominantly later-generation devices (Evolut R: 2.0%, PRO: 36.7%, and PRO+: 61.2%; χ^2^ = 28.3, *p* < 0.001), confirming that technique and device generation are strongly collinear in this cohort.

### 3.3. Primary and Secondary Endpoints—Unmatched Cohort

**PPMI (Primary Endpoint):** A total of 3 patients (6.1%) in the COT group vs. 6 patients (9.7%) in the three-cusp group (*p* = 0.48) (Figure 1).

**Secondary Composite Endpoint:** In total, 14.30% of patients in the cusp overlap group vs. 32.30% in the three-cusp group (*p* = 0.049) (Figure 2). Individual components are reported in the Supplementary Materials, Table S2; the difference is driven predominantly by the moderate/severe PVL component (24.2% vs. 12.2%), and, to a lesser extent, 30-day death (4.8% vs. 0%), rather than by stroke (1.6% vs. 0%) or major vascular complications (4.8% vs. 2.0%), none of which differed significantly between arms individually.

**Complications:** There were two cases of intraprocedural death due to valve embolization in the three-cusp group (1.76%). Moderate paravalvular regurgitation occurred more frequently in the three-cusp group (24% vs. 12.2%; *p* = 0.17) (Figure 3).

### 3.4. Propensity Score—Matched Cohort

Forty-nine pairs of patients were analyzed after propensity score matching. After matching, no significant differences were found for age, aortic annulus diameter, or other baseline characteristics (Table 3). Pre-matching standardized mean differences for the covariates entered into the propensity model, computed directly from the study database, are reported in the Supplementary Materials, Table S1. A temporal analysis of PPMI, PVL, and technique/device-generation distribution by implantation year is presented in the Supplementary Materials, Table S1, to explore confounding from the operator learning curve and device generation.

### 3.5. Temporal/Sequential Analysis

COT adoption increased markedly over the study period, confirming the learning-curve/calendar-time confounding raised in the review. By implantation year: 2020—0/11 COT (0%); 2021—12/53 COT (22.6%); 2022—31/41 COT (75.6%); 2023—6/6 COT (100%). This calendar-time shift parallels the device-generation shift noted above (earlier years dominated by Evolut R/PRO, later years by PRO+), so technique, operator experience, and device generation cannot be fully disentangled in this single-center cohort (Table 3).

In the unmatched cohort, the incidence of new PPMI was 6.1% in the cusp overlap group versus 9.7% in the three-cusp group (*p* = 0.48). Consistent results were also observed in the matched cohort (COT: 6.1% vs. 3CT: 12.2%; *p* = 0.48) (Figure 4).

## 4. Discussion

In this retrospective analysis of 113 patients undergoing TAVR with the third-generation Medtronic Evolut™ SE-THV, we did not find a statistically significant reduction in PPMI rates using the cusp-overlap technique. PPMI occurred in 6.1% of patients in the COT group versus 9.7% (unmatched cohort) and 12.2% (propensity score-matched cohort), respectively (*p* = 0.48). Given the low absolute number of primary endpoint events (3 vs. 6 in the matched cohort), the post hoc power to detect this difference was approximately 19% at α = 0.05; the negative result should, therefore, be interpreted as inconclusive rather than as evidence that COT and 3CT produce equivalent PPMI rates. However, COT was associated with a numerically lower incidence of moderate or severe paravalvular regurgitation (PVL) and a significantly reduced rate of the composite secondary endpoint of adverse clinical events at 30 days (unmatched: 14.3% vs. 32.3%; matched: 14.3% vs. 30.6%, corrected from the 32.3% used for the matched cohort in the original submission; *p* = 0.049), without prolonging procedural time. As detailed above, this composite result is driven predominantly by the PVL component and, secondarily, by 30-day death, rather than equally by all hard clinical events, and should be regarded as hypothesis-generating.

These findings must be interpreted in the context of the evolving evidence regarding implantation techniques for self-expanding transcatheter heart valves (SE-THVs). Conduction disturbances remain one of the most frequent complications after TAVR with SE platforms, primarily driven by mechanical interaction between the valve frame and the conduction system in the membranous septum [21,22]. Implantation depth (ID) and the relationship to patient-specific anatomy (particularly membranous septum length—MSL) are key modifiable factors [23,24]. The COT, first systematically described by Tang et al. [14], improves visualization of the non-coronary cusp (NCC) and allows for more precise, higher deployment relative to the left ventricular outflow tract, which is theorized to minimize trauma to the atrioventricular node and His bundle.

Early single- and multi-center observational studies consistently suggested a benefit of COT [25,26]. Pascual et al. reported a reduction in the new PPMI rates from 23% to 12.4% (*p* = 0.037) [13], later confirmed in a propensity-matched analysis (11.8% vs. 21.7%; *p* = 0.03) [27]. Doldi et al. observed a decrease from 27.9% to 13.1% (*p* = 0.047) [16], while Mendiz et al. demonstrated an even more pronounced effect (30.9% vs. 6.5%; *p* = 0.0001) [28]. Similar trends were reported by other groups, with COT achieving higher implantation positions (typically 1–2 mm shallower when measured from the NCC) and lower rates of high-degree atrioventricular block [27].

Our study aligns with this body of evidence in several important aspects, yet differs in the magnitude of the PPMI benefit. The absolute PPMI rate in the COT arm (6.1%) is among the lowest reported in real-world cohorts using the Evolut platform, and is comparable to the best-performing centers in the Optimize PRO study (6.4–9.8% when in high compliance with a refined four-step COT protocol). This low event rate likely reflects progressive operator experience, meticulous pre-procedural CT planning, and a conservative post-procedural conduction monitoring strategy in line with expert consensus documents. It may also partly reflect the correlation between later COT adoption and the later, lower-PPMI device generations employed in our cohort.

The absence of statistical significance for the primary endpoint reflects the limited sample size and low event count, formally quantified above as ~19% post hoc power; no a priori sample size calculation was performed, which we acknowledge as a methodological limitation. The numerical reduction (an approximately 50% relative risk reduction in the matched cohort) is directionally concordant with larger series, but given the wide, overlapping confidence intervals around our estimates (Supplementary Materials, Table S2), it cannot be considered confirmatory evidence of clinical benefit on its own.

Recent larger-scale data have further strengthened the case for COT. A multi-center analysis of over 2200 patients treated with the Evolut platform (Wiennemann et al. [29]) confirmed a significant reduction in PPMI (12.3% COT vs. 17.0% 3CT; *p* = 0.002) and moderate/severe PVL after propensity matching, with COT emerging as an independent protective factor (OR: 0.63). Two contemporary meta-analyses encompassing over 5000 patients have quantified the benefit: COT was associated with a 37–44% relative reduction in PPMI (OR/RR: 0.53–0.63), lower rates of complete AV block and new left bundle branch block, shorter hospital stays, and improved hemodynamic parameters [30,31]. In the Optimize PRO study (including the FX addendum), systematic use of refined COT with the Evolut Pro/FX platform yielded PPMI rates as low as 6.7% at 30 days (and 8.8% at 1 year) when protocol compliance was high, together with near-zero moderate/severe PVL and a median length of stay of 1 day [32].

Mechanistically, the advantage of COT appears to stem not only from achieving a higher absolute ID, but from better alignment with patient-specific anatomy. While some studies (including a recent propensity-weighted analysis by Lemarchand et al. [33]) suggest that COT itself may not be independently associated with reduced conduction disturbances after comprehensive adjustment for baseline ECG abnormalities and valve type, it consistently facilitates a more controlled and shallower deployment relative to the membranous septum. In our cohort, the lower incidence of moderate/severe PVL with COT may be explained by the conical design of the Evolut valve, which exerts greater radial force at the annular level when positioned higher, combined with improved co-axiality and reduced rocking during deployment.

Importantly, COT did not increase procedural complexity in our experience. Procedure time was numerically shorter in the COT group (a median of 110 min in both arms, with a lower mean and narrower spread in the COT group), consistent with reports indicating a rapid learning curve once the optimal projection is planned on pre-procedural CT. This reinforces that COT can be safely integrated into routine practice without additional resource utilization, although we note that operator experience and device generation evolved concurrently with technique adoption over the study period.

### Study Limitations

This is an observational study with inherent limitations, mainly selection and confounding biases, which we attempted to reduce by propensity score analysis. Caution must be taken when extrapolating the results given the lack of an optimal sample size.

The study was not accompanied by an a priori sample size calculation, and the post hoc power for the primary endpoint (PPMI) was approximately 19% at α = 0.05; the negative primary result should, therefore, be considered inconclusive rather than evidence of equivalence between COT and 3CT.

Prosthetic valve implantation depth and membranous septum length were not measured or calculated in this cohort, for any patient, either at the time of the procedure or retrospectively. Because the theoretical mechanism of COT rests specifically on achieving a shallower, more controlled implant relative to the conduction system, the complete absence of these measurements means this study cannot confirm that COT engaged its intended mechanism at all. All mechanistic interpretations offered in Section 4 are drawn from the cited literature and should be regarded as hypotheses, not as findings demonstrated by our own data.

Baseline conduction status—pre-existing right bundle branch block, first-degree atrioventricular block, and baseline QRS/PR duration—was not consistently recorded for all patients in this retrospective database and could not be reported as a complete comparison between the COT and 3CT groups, nor entered into the propensity score model. Undetected imbalance in baseline conduction status between the two groups therefore cannot be excluded, and this should be considered when interpreting the PPMI and PVL findings.

Patients were not randomized to COT versus 3CT; COT cases were concentrated later in the study period (0% of cases in 2020, rising to 100% in 2023—see the temporal analysis), introducing confounding from the operator learning curve, evolving patient selection, and device generation, which was confirmed to differ significantly by technique (Evolut R/PRO predominating in 3CT, PRO+ predominating in COT; χ^2^ *p* < 0.001). Propensity matching adjusted for baseline patient characteristics but did not include device generation or calendar time, and could not fully separate the effect of technique from these correlated temporal factors.

The secondary composite endpoint combines hard clinical events with an echocardiography-based imaging parameter (PVL); as shown in the Supplementary Materials, Table S2, the composite result was driven predominantly by the PVL component and, secondarily, by 30-day death, rather than by stroke or major vascular complications. PVL was graded by the treating cardiologist rather than by a blinded core laboratory, introducing potential inter-observer variability.

Finally, the study focused on a particular type of self-expanding valve (Medtronic Evolut™), and the follow-up period was limited to 30 days.

## 5. Conclusions

In this single-center real-world cohort, the cusp-overlap technique was not associated with a statistically significant reduction in PPMI after TAVR with the CoreValve Evolut™ self-expanding valve; this negative primary result should be interpreted with caution given the limited event count. COT was associated with fewer paravalvular leaks and a lower rate of a composite secondary endpoint driven predominantly by the PVL component; these secondary findings are hypothesis-generating rather than confirmatory. Future randomized controlled trials—adequately powered for PPMI and incorporating standardized implantation-depth measurement and complete baseline conduction assessment—are needed to confirm these benefits and further standardize TAVR protocols in clinical practice.

## Figures and Tables

**Figure 1:**
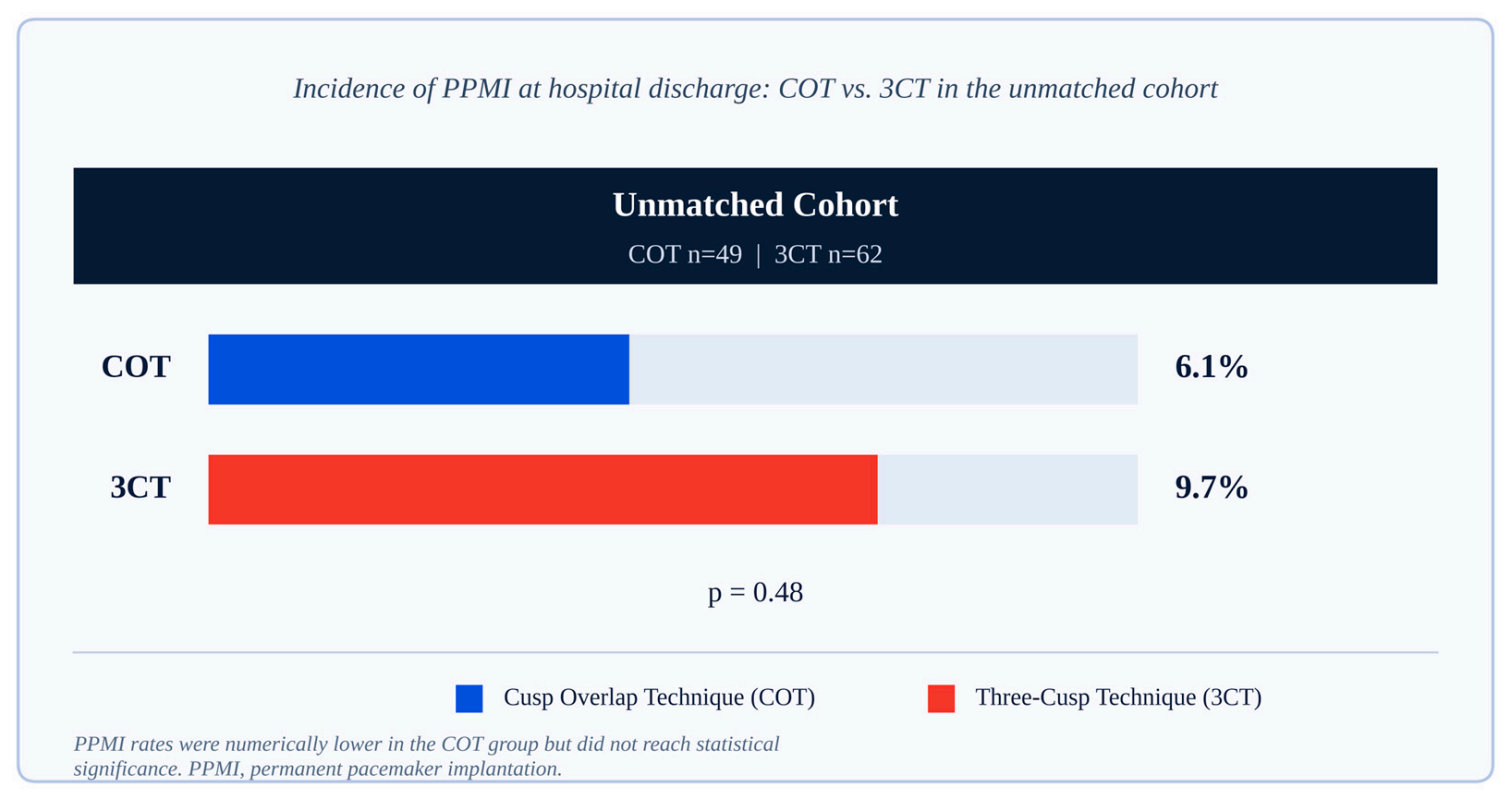
Primary endpoint–PPMI at hospital discharge–unmatched cohort.

**Figure 2:**
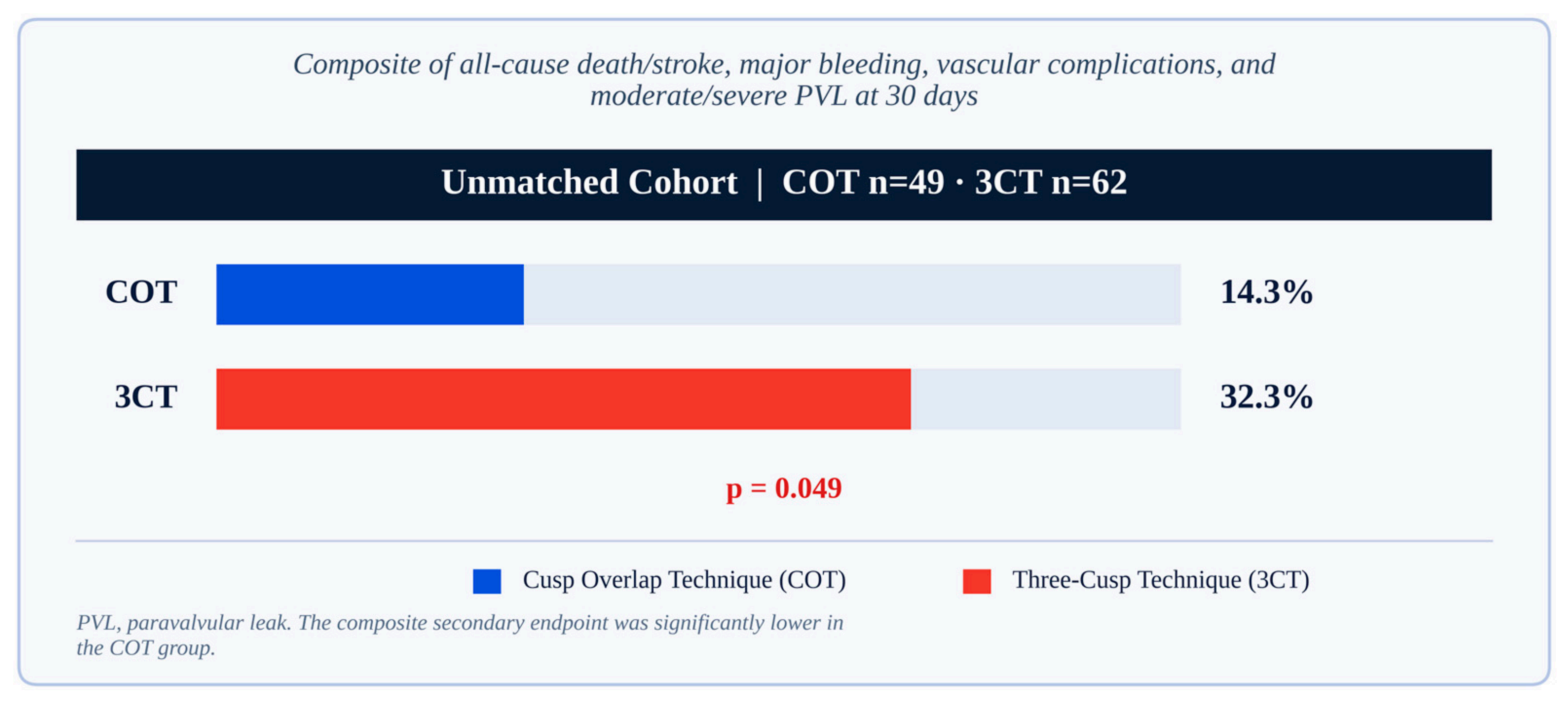
Secondary composite endpoint–unmatched cohort.

**Figure 3:**
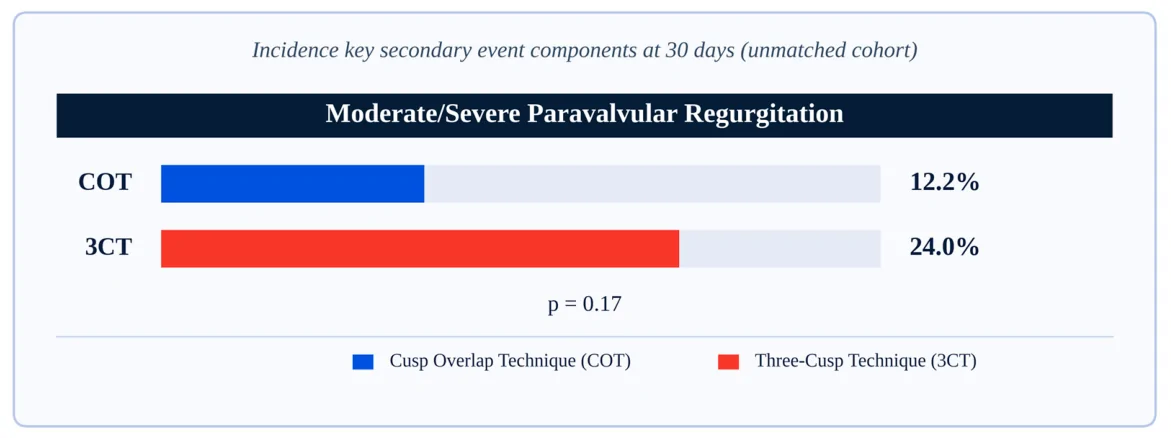
Moderate/severe paravalvular regurgitation–unmatched cohort.

**Figure 4:**
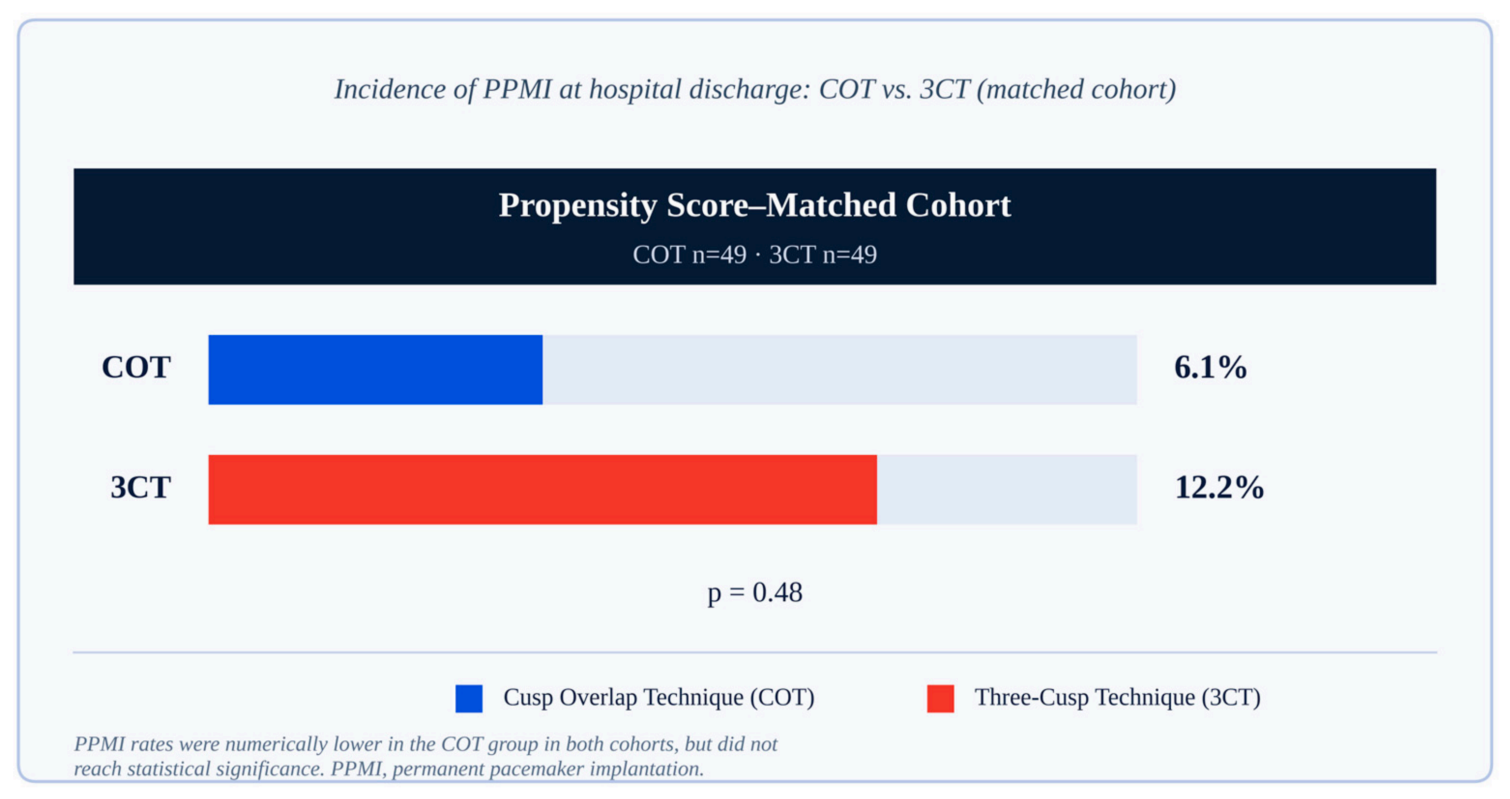
Primary endpoint–propensity score-matched cohort.

**Table 1:** Baseline characteristics—unmatched cohort. Data expressed as n (%) or median (IQR). CABG, coronary artery bypass grafting; NYHA, New York Heart Association; STS, Society of Thoracic Surgeons.

Variable	3CT (n = 62)	COT (n = 49)	*p* Value
Age, years	84 (79.25; 88)	84 (80.75; 87)	0.837
Women, n (%)	38 (61.3)	32 (65.3)	0.812
Hypertension	39 (62.9)	30 (61.2)	1.00
Hemoglobin (g/dL)	12 (11.1; 13.3)	12.4 (11.5; 13.8)	0.182
Hyperlipidemia	39 (62.9)	25 (51.0)	0.287
Smoking	11 (17.7)	15 (30.6)	0.173
Diabetes mellitus	13 (21.0%)	8 (16.3%)	0.707
Coronary artery disease	32 (51.6)	16 (32.7)	0.070
Previous stroke	1 (1.6)	2 (4.1)	0.836
Previous CABG	7 (11.3)	5 (10.2)	1.00
Renal insufficiency	29 (46.8)	12 (24.5)	0.027
Peripheral artery disease	8 (12.9)	10 (20.4)	0.420
NYHA class III or IV	26 (42.0)	19 (38.8)	0.419
STS risk score, %	8 (5.00; 11.25)	8 (6.0; 12)	0.523
Aortic annulus diameter (mm)	20 (19; 22)	20 (19; 21)	0.618

**Table 2:** Evolut device generation by technique (unmatched cohort, n = 111). Device generation extracted from the procedural database (valve type field) and cross-tabulated with technique. No Evolut FX cases were identified in this cohort (2020–2023 predates FX adoption at our center).

Device Generation	3CT (n = 62)	COT (n = 49)	χ^2^ = 28.3, df = 2
Evolut R	20 (32.3%)	1 (2.0%)	*p* < 0.001
Evolut PRO	31 (50.0%)	18 (36.7%)	
Evolut PRO+	11 (17.7%)	30 (61.2%)	

**Table 3:** COT adoption over the study period.

Year	n (Total)	COT, n (%)	PPMI, 3CT/COT	PVL, 3CT/COT	Dominant Generation
2020	11	0 (0%)	1/11 (9.1%)/—	1/11 (9.1%)/—	Evolut R
2021	53	12 (22.6%)	2/41 (4.9%)/0/12 (0%)	12/41 (29.3%)/1/12 (8.3%)	Evolut PRO
2022	41	31 (75.6%)	3/10 (30.0%)/3/31 (9.7%)	2/10 (20.0%)/3/31 (9.7%)	Evolut PRO/PRO+
2023	6	6 (100%)	—/0/6 (0%)	—/2/6 (33.3%)	Evolut PRO+

Derived from procedure dates (DOP field) in the procedural database, cross-tabulated with technique, PPMI, and PVL outcomes for the n = 111 unmatched analytic cohort (two intraprocedural deaths excluded). Cell counts are small in some years (e.g., 2023, n = 6), so year-specific rates should be interpreted cautiously and are presented descriptively rather than with formal between-year testing.

## Data Availability

Data can be obtained from the Monaco Cardiothoracic Center.

## Supplementary Materials

The following supporting information can be downloaded at: https://www.mdpi.com/article/10.3390/jcm15197413/s1, Table S1: Pre-matching standardized mean differences (SMD), unmatched cohort; Table S2: Secondary composite endpoint–individual components (95% CI); Table S3. Baseline characteristics—propensity score–matched cohort. Data expressed as n (%) or median (IQR).
