# Supplementary material for: The Cusp-Overlap Technique Versus the Standard Three-Cusp Technique for Self-Expanding Transcatheter Aortic Valves—A Single-Center Experience
Source: J Clin Med. 2026 Sep 24;15(19):7413. doi: 10.3390/jcm15197413 (PMC13666216; doi:10.3390/jcm15197413)
Supplement: Supplementary file 1 [file jcm-15-07413-s001.zip › jcm-4380011-supplementary.pdf]

Supplementary Table S1. Pre-matching standardized mean differences (SMD), unmatched cohort.

Covariate	3CT (n=62)	COT (n=49)	SMD
Age, years (mean)	82.8	83.5	-0.10
STS risk score, % (mean)	8.6	9.5	-0.16
Renal insufficiency, %	46.8	24.5	0.48
Coronary artery disease, %	51.6	32.7	0.39
Hyperlipidemia, %	62.9	51.0	0.24
Smoking, %	17.7	30.6	-0.30
Hypertension, %	62.9	61.2	0.04
Peripheral artery disease, %	12.9	20.4	-0.20
NYHA class III/IV, %	41.9	38.8	0.06

SMD computed as ($mean_3CT$ − meanCOT)/pooled SD for continuous variables and (p_3CT − pCOT)/$\sqrt{[(p_3CT(1-p_3CT)+pCOT(1-pCOT))/2]}$ for binary variables, from the pre-matching (unmatched) cohort database. Renal insufficiency (SMD 0.48) and coronary artery disease (SMD 0.39) show the largest pre-matching imbalance, consistent with the significant unmatched p-value for renal insufficiency in Table 1 and motivating the propensity match. Post-matching SMDs are not available (see text).

Supplementary Table S2. Secondary composite endpoint – individual components (95% CI).

Component	3CT, n (%)	COT, n (%)	95% CI (3CT / COT)	Cohort
30-day death	3 (4.8%)	0 (0.0%)	1.7–13.3 / 0.0–7.3	Unmatched
Stroke	1 (1.6%)	0 (0.0%)	0.3–8.6 / 0.0–7.3	Unmatched
Major vascular complications	3 (4.8%)	1 (2.0%)	1.7–13.3 / 0.4–10.7	Unmatched
Moderate/severe PVL	15 (24.2%)	6 (12.2%)	15.2–36.2 / 5.7–24.2	Unmatched
**Composite (≥1 event)**	**20 (32.3%)**	**7 (14.3%)**	**22.0–44.6 / 7.1–26.7**	**Unmatched**
30-day death	3 (6.1%)	0 (0.0%)	2.1–16.5 / 0.0–7.3	Matched
Major vascular complications	2 (4.1%)	1 (2.0%)	1.1–13.7 / 0.4–10.7	Matched
Moderate/severe PVL	11 (22.4%)	6 (12.2%)	13.0–35.9 / 5.7–24.2	Matched
**Composite (≥1 event)**	**15 (30.6%)**	**7 (14.3%)**	**19.5–44.5 / 7.1–26.7**	**Matched**

Unmatched-cohort rows (n=62 3CT, n=49 COT) are exact patient-level counts, independently recomputed from the anonymized outcomes database and cross-validated against the composite total already published (32.3%/14.3%: exact match). Matched-cohort rows reproduce the statistician's returned aggregate percentages (Sheet4/Sheet5 of the file sent for propensity matching), converted to counts (rounded) and Wilson 95% CIs; per-patient matched-pair data were not available to recompute these directly. ***The matched-cohort composite of 30.6% (highlighted) corrects the value of 32.3% used for the matched cohort in the original submission (Figure 6/Discussion), which appears to have been copied from the unmatched-cohort value.***

Table S3. Baseline characteristics—propensity score–matched cohort. Data expressed as n (%) or median (IQR).

Variable	3CT (n = 49)	COT (n = 49)	*p* Value
Age, years	84 (80.5; 87)	84 (79.25; 88)	0.781
Women, n (%)	31 (63.3)	32 (65.3)	1.00
Hypertension	29 (59.2)	30 (61.2)	1.00
Hemoglobin (g/dL)	12.2 (11.1; 13.5)	12.4 (11.5; 13.8)	0.359
Hyperlipidemia	31 (63.3)	25 (51.0)	0.307
Smoking	9 (18.4)	15 (30.6)	0.240
Diabetes mellitus	8 (16.3)	8 (16.3)	1.00
Coronary artery disease	22 (44.9)	16 (32.7)	0.30
Prior stroke	1 (2.0)	2 (4.1)	1.00
Prior CABG	5 (10.2)	5 (10.2)	1.00
Chronic kidney disease	21 (42.9)	12 (24.5)	0.087
Peripheral artery disease	8 (12.9)	10 (20.4)	0.794
NYHA class III or IV	23 (46.9)	19 (38.8)	0.649
STS risk score, %	8 (6.0; 12)	8 (6.0; 12)	0.906
Aortic annulus diameter (mm)	22 (20; 23)	21 (20; 23.75)	0.868

After propensity score matching, all baseline characteristics were well-balanced between groups. CABG, coronary artery bypass grafting; NYHA, New York Heart Association; STS, Society of Thoracic Surgeons.
